# Ventilatory Limitation of Exercise in Pediatric Subjects Evaluated for Exertional Dyspnea

**DOI:** 10.3389/fphys.2019.00020

**Published:** 2019-01-29

**Authors:** Paolo T. Pianosi, Joshua R. Smith

**Affiliations:** ^1^Department of Pediatric and Adolescent Medicine, Mayo Clinic, Rochester, MN, United States; ^2^Department of Cardiovascular Medicine, Mayo Clinic, Rochester, MN, United States

**Keywords:** flow-volume curve, flow limitation, ventilation, dyspnea, exercise, children

## Abstract

**Purpose:** Attribution of ventilatory limitation to exercise when the ratio of ventilation (${\dot{V}}_{E}$) at peak work to maximum voluntary ventilation (MVV) exceeds 0.80 is problematic in pediatrics. Instead, expiratory flow limitation (EFL) measured by tidal flow-volume loop (FVL) analysis – the method of choice – was compared with directly measured MVV or proxies to determine ventilatory limitation.

**Methods:** Subjects undergoing clinical evaluation for exertional dyspnea performed maximal exercise testing with measurement of tidal FVL. EFL was defined when exercise tidal FVL overlapped at least 5% of the maximal expiratory flow-volume envelope for > 5 breaths in any stage of exercise. We compared this method of ventilatory limitation to traditional methods based on MVV or multiples (30, 35, or 40) of FEV_1_. Receiver operating characteristic curves were constructed and area under curve (AUC) computed for peak ${\dot{V}}_{E}$/MVV and peak ${\dot{V}}_{E}$/*x*⋅FEV_1_.

**Results:** Among 148 subjects aged 7–18 years (60% female), EFL was found in 87 (59%). Using EFL shown by FVL analysis as a true positive to determine ventilatory limitation, AUC for peak ${\dot{V}}_{E}$/30⋅FEV_1_ was 0.84 (95% CI 0.78–0.90), significantly better than AUC 0.70 (95% CI 0.61–0.79) when 12-s sprint MVV was used for peak ${\dot{V}}_{E}$/MVV. Sensitivity and specificity were 0.82 and 0.70 respectively when using a cutoff of 0.85 for peak ${\dot{V}}_{E}$/30⋅FEV_1_ to predict ventilatory limitation to exercise.

**Conclusion:** Peak ${\dot{V}}_{E}$/30⋅FEV_1_ is superior to peak ${\dot{V}}_{E}$/MVV, as a means to identify potential ventilatory limitation in pediatric subjects when FVL analysis is not available.

## Introduction

The ventilatory response to exercise changes through childhood and adolescence (Cooper et al., 1987). Younger children have higher ventilatory equivalents (Armstrong et al., 1997; Prioux et al., 1997) such that they achieve levels of ventilation (${\dot{V}}_{E}$) near maximum voluntary ventilation (MVV) at peak work (Godfrey, 1974); but this morphs through adolescence until peak ${\dot{V}}_{E}$/MVV ratio reaches typical adult levels (Rowland and Cunningham, 1997; Giardini et al., 2011). Pre-pubertal children develop significant expiratory flow limitation (EFL) during exercise (Nourry et al., 2006; Swain et al., 2010; Borel et al., 2014) with the prevalence of EFL decreasing post-puberty due at least in part to lower ventilatory requirement (Emerson et al., 2015; Smith et al., 2015). It behooves one to understand EFL during exercise as it may limit ${\dot{V}}_{E}$, worsen dyspnea, or reduce capacity (Dempsey et al., 2008; Babb, 2013).

Cardiopulmonary exercise testing is used clinically to investigate exertional dyspnea. The traditional method of assessing ventilatory limitation is based on breathing reserve (Ross, 2003):

$$100\%\cdot [1-\mathrm{peak}{\dot{V}}_{E}/\mathrm{MVV}]$$

This approach is problematic as it ignores the fact that the maximal voluntary ventilation (MVV) maneuver does not mimic the breathing pattern or respiratory mechanics that occur during exercise (Klas and Dempsey, 1989; Agostoni et al., 2011). Prevailing wisdom in pediatric exercise medicine maintains that children achieve “near to or slightly less than 70% of their MVV at maximal ventilation” (Orenstein, 1993; Takken et al., 2017) but this observation has never been directly tested. Furthermore, proxy measures for MVV based on multiples of FEV_1_ are often used rather than direct measurement of MVV but *only* 35⋅FEV_1_ has been examined in a pediatric population. Specifically, Fulton et al. (1995) found that MVV was similar to the proxy measure of MVV viz 35⋅FEV_1_ in healthy African–American, adolescent girls. Furthermore, a recent study found that conclusions from test results depend on which surrogate for MVV is chosen only compounds interpretation challenges (Colwell and Bhatia, 2017). Taken together, it is not at all clear whether directly measured MVV or multiples of FEV_1_ as proxies for MVV reflect EFL and therefore ventilatory limitation during exercise in children and adolescents.

Thus, the central question of this study is whether a simple, reproducible test that incorporates confounding and inter-related variables such as age, sex, and height, enables one to confidently identify whether a subject would exhibit EFL during exercise. Tidal flow-volume loop (FVL) analysis was used as the method of choice to confirm EFL during exercise and compare with directly measured MVV vs. multiples of FEV_1_ as proxies for MVV. We hypothesized that a proxy measure of MVV based on FEV_1_ predicts development of EFL and ventilatory limitation during exercise in pediatric subjects undergoing testing for investigation of exertional dyspnea.

## Materials and Methods

### Subjects

Medical records of children and adolescents up to 18 years of age seen at Mayo Clinic from 2007 to 2014, who underwent clinically indicated, maximal exercise tests with FVL analysis as part of clinical evaluation of exertional dyspnea were audited retrospectively. Subjects with exercise-induced laryngeal obstruction that was diagnosed by continuous laryngoscopy during exercise were excluded. The cohort was comprised of patients with known asthma who still complained of exertional dyspnea despite aggressive therapy, patients with disease in other organ systems affecting the respiratory system, and subjects evaluated for exertional dyspnea or chest pain in which no cause was found and thus had no specific medical diagnosis. Diagnosis of asthma required a history of compatible symptoms plus evidence of airway hyperreactivity or bronchodilator responsiveness. Patients with asthma were clinically stable when tested. Informed consent was not required as testing was conducted for clinical indications. Minnesota statute permits retrospective chart review for an IRB approved protocol. Mayo Clinic Institutional Review Board approved the study with waiver of consent.

### Pulmonary Function Tests

Routine instructions for pulmonary function testing (PFTs) included avoiding short-acting bronchodilator for at least 4 h and long-acting bronchodilators for at least 12 h. All subjects performed PFTs immediately prior to exercise on the same MedGraphics system used for the exercise test (see below) according to ATS/ERS criteria (Miller et al., 2005). Subjects performed PFTs while seated on the cycle ergometer and their largest maximum expiratory flow-volume envelope was used for analysis. PFTs were expressed as percent of predicted (Knudson et al., 1983). MVV was measured in all subjects by the 12-s sprint method on a Jaeger MasterScreen, on the same day as exercise or within the same week (median [IQR] time 0 [0–4] days) in a subset of subjects undergoing bronchoprovocation challenge (to complete workup for exertional dyspnea). Post exercise PFTs were performed only at the discretion of the triage physician, as all testing was done for clinical indications.

### Exercise Test

Subjects were instructed to fast 2 h before the test. They performed a maximal exercise test on a Corival V3 cycle ergometer. We employed James’ protocol consisting of three programs for three ranges of body surface area starting at 200 kg m min^-1^ with increments of 100 or 200 kg m min^-1^ every 3 min depending on body surface area prior to 2008 (James, 1981). All subsequent tests were done using Godfrey protocol (Godfrey, 1974) starting at 10 to 25 W, with step increments of 10 to 25 W min^-1^ based on subject’s height and sex, in order to obtain test duration of 10 ± 2 min. Patients were strongly encouraged to exercise to volitional fatigue in order to achieve criteria (e.g., gas exchange ratio > 1.1, HR > 190 bpm) implying maximal effort. Heart rate and SpO_2_ were monitored continuously with a 12-lead ECG and pulse oximetry, respectively. Blood pressure was measured every other workload.

### Ventilatory Measurements During Exercise

Ventilation and gas exchange were measured breath-by-breath via MedGraphics CPX/D (Breeze software) that employs a Pitot tube to measure flow, electronically integrated to yield volume. The software corrects for drift that occurs when inspiratory and expiratory volumes differed. Exhaled gasses were measured by mass spectrometry. System calibration was done prior to every exercise test. The Breeze© program measures EFL according to method described by Johnson et al. (1995) at Mayo Clinic. In short, the degree of EFL was obtained by aligning a tidal breath during exercise within the maximum expiratory flow-volume curve. Alignment was achieved by having subjects perform an inspiratory capacity (IC) maneuver from end-expiratory lung volume. The program permits review of individual FVLs on a breath by breath basis, and automatically computes percent overlap of tidal breath with maximum expiratory FVL. ERV expiratory reserve volume was calculated by subtracting IC from forced vital capacity. IC maneuvers were rehearsed prior to exercise until subjects demonstrated acceptable consistent maneuvers. Once exercise began, an IC maneuver was repeated during a 3-min warm-up at the first workload, then every other load (alternating with blood pressure) until the respiratory compensation point, after which most subjects were able to perform only 1–2 more loads before blood pressure check at peak exercise.

### Definition of EFL

Consensus definition of EFL has not yet been formulated. Nourry et al. (2005) defined EFL to occur when “part of the exercise FVL met the boundary of the expiratory portion of the maximum expiratory FVL determined before exercise” and considered subjects to be flow limited when EFL was observed over ≥ 5% exercise tidal volume, maintained up to peak work. Swain et al. (2010) similarly defined EFL when intersection of the exercise tidal loop and maximal FVL was >5% for any breath. We defined EFL as ≥5% tidal volume overlap with the maximum expiratory flow-volume envelope for >5 breaths during sub-maximal or peak exercise.

### Statistical Analysis

Comparisons between subjects with and without EFL were evaluated using two-sample *t-* or χ^2^ tests as appropriate. Different multiples (*x*) of FEV_1_ were calculated and predictive ability using area under curve (AUC) was computed from ROC curves plotted for peak ${\dot{V}}_{E}$/MVV and peak ${\dot{V}}_{E}$/*x*⋅FEV_1_, using presence of EFL as the method of choice to determine exercise limited by ventilation. Optimal cutoff was chosen as the value with highest sensitivity and specificity using the point closest to perfect separation. This analysis was done using R software v. 3.2.3, with significance set at *p* < 0.05. We dichotomized subject into flow limited and non-flow limited, such that EFL was a fixed factor and workload was fitted as a within-subjects factor, to analyze behavior of operating lung volume during exercise between subjects with vs. without EFL. Changes in operating lung volume were calculated as change from rest in expiratory reserve volume (ERV) and IC as fractions of a one’s VC; i.e., ERV/VC and IC/VC. As these were not measured at every workload, they were binned according to relative exercise intensity: 8–19, 20–55, and 55–90% peak workload for analysis. A mixed effects regression model was fit to assess the effect of EFL on ERV/VC and on IC/VC at different workloads using Stata v.14.0.

## Results

### Subjects

The final sample comprised 148 subjects: mean ± SD age 14.3 ± 2.6 years, height 164 ± 13 cm, and weight 59.3 ± 15.5 kg. Eighty-six (58%) subjects presented as exertional dyspnea (DoE) with no underlying disorder, and 38 (26%) had asthma. The remaining 24 (16%) subjects (“other”) comprised congenital heart disease (*n* = 7), pectus excavatum (*n* = 4), colitis (*n* = 2), bronchiectasis (*n* = 2), cardiac dysrhythmia *n* = 2), plus one subject each with mediastinal fibrosis, postural tachycardia syndrome, CF-related metabolic syndrome, scoliosis with fused ribs, weakness, angioedema, and post-ARDS, presenting with dyspnea or chest pain on exertion. Lung function data are shown in Table 1.

**Table 1:** Pulmonary function test results, means (SD), split by EFL.

	No EFL	EFL	*p*-value^∗^
FVC (%pred)	105 (15)	101 (17)	0.16
FVC (L)	3.94 (1.05)	3.85 (1.20)	0.65
FEV_1_ (%pred)	103 (15)	94 (17)	0.0007
FEV_1_ (L)	3.97 (1.06)	3.32 (0.59)	0.09
FEV_1_/FVC	0.86 (0.07)	0.88 (0.08)	<0.0001
FEF_50_ (%pred)	81 (23)	64 (22)	<0.0001
FEF_50_ (L⋅s^-1^)	4.09 (1.20)	3.39 (1.26)	0.0008
MVV (L⋅min^-1^)	93 (28)	98 (37)	0.37


*^∗^Unpaired *t-*tests.*

### EFL During Exercise

Expiratory flow limitation occurred at some point during exercise in 87 (59%) subjects, over 49 ± 21% (mean ± SD) of tidal volume. Onset of EFL during exercise occurred in lighter exercise in those with more obstruction (greater concavity of resting maximum expiratory FVL) but not until peak exercise in those with normal spirometry (Pianosi, 2018b). Subjects with EFL achieved higher values for peak $\dot{V}O_{2}$ with concomitant higher minute volume and O_2_ pulse at peak exercise (Table 2). No subjects exhibited SpO_2_< 94%.

**Table 2:** Peak exercise results in each sex stratified by EFL.

	Females		Males	
		
Mean ± SD	No EFL (*N* = 43)	EFL (*N* = 46)	No EFL (*N* = 18)	EFL (*N* = 41)
Age (years)	14.9 ± 2.0	14.7 ± 2.8	13.6 ± 3.5	13.7 ± 2.6
Height (cm)	163.4 ± 7.6	160.7 ± 12.1	165.7 ± 20.2	166.2 ± 15.6
Weight (kg)	58.3 ± 10.7	57.0 ± 13.4	59.5 ± 19.7	62.8 ± 19.9
BMI (kg⋅m^2^)	21.8 ± 3.5	21.8 ± 4.0	21.1 ± 4.6	22.3 ± 4.6
BSA (m^2^)	1.62 ± 0.16	1.59 ± 0.23	1.65 ± 0.37	1.69 ± 0.32
Work (W)	144 ± 37	160 ± 56	173 ± 70	185 ± 69
Work (%pred^∗^)	88 ± 23^†^	102 ± 24^†^	77 ± 19	90 ± 25
$\dot{V}O_{2}$ (mL⋅kg^-1^⋅min^-1^)	33.1 ± 6.1^†^	36.2 ± 7.5^†^	39.0 ± 7.3	39.5 ± 9.2
$\dot{V}O_{2}$ (%predicted^∗^)	89.5 ± 18.0	99.1 ± 19.4	85.4 ± 16.1	88.3 ± 17.5
HR (beat⋅min^-1^)	184 ± 14	185 ± 11	185 ± 15	185 ± 13
O_2_ pulse (mL⋅beat)	10.4 ± 2.3	11.1 ± 3.3	12.3 ± 41	13.2 ± 4.3
RR (breath⋅min^-1^)	47 ± 9^‡^	53 ± 9^‡^	55 ± 16	54 ± 9
VT (L)	1.55 ± 0.33	1.58 ± 0.52	1.75 ± 0.91	1.77 ± 0.66
${\dot{V}}_{E}$ (L⋅min^-1^)	69.7 ± 16.1^ 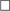 ^	82.3 ± 25.0^ 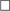 ^	84.5 ± 32.2	93.1 ± 34.6
${\dot{V}}_{E}$ /$\dot{V}O_{2}$	37.6 ± 5.9	40.2 ± 4.8	37.3 ± 5.8	38.1 ± 6.1
RER	1.15 ± 0.08	1.20 ± 09	1.16 ± 0.12	1.19 ± 0.10
PetCO_2_ (mmHg)	35 ± 4	34 ± 4	35 ± 4	36 ± 4


*^∗^Godfrey for Work Godfrey (1974) and Cooper et al. (1984). ^†^*p* ≤ 0.006, ^‡^*p* = 0.001, ^
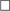
^*p* = 0.01 (*t*-test).*

**FIGURE 1:**
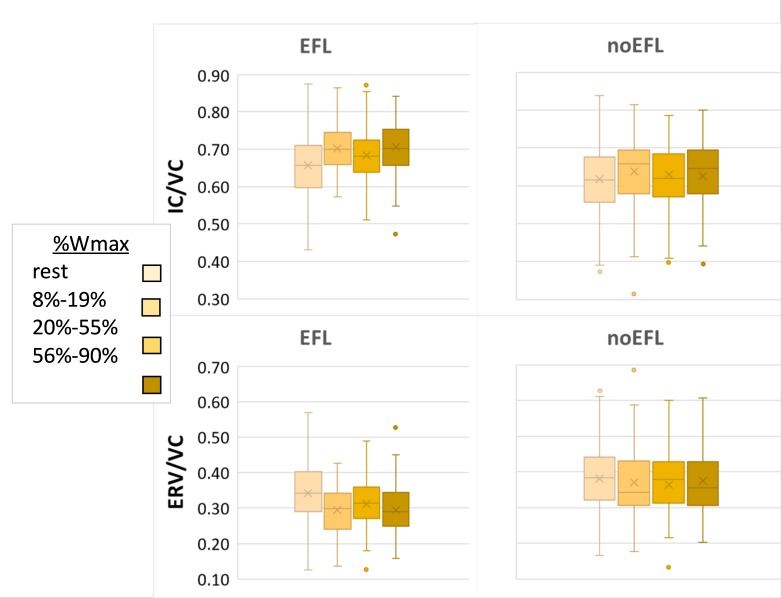
Operating lung volume. Box and whisker plots of operating lung volume during exercise, expressed as ratio of inspiratory capacity (IC) or expiratory reserve volume (ERV) divided by vital capacity (VC), in subjects according to presence or absence EFL in exercise. Box represents 25^th^ and 75^th^ centiles, with horizontal line representing median and x depicting mean value; dots are outliers.

All subjects augmented tidal volume at onset of exercise by decreasing ERV. The effect of work level on ERV/VC did not show a linear trend as the largest change occurred between rest and light exercise, mirrored by a rise in IC/VC. Those who developed EFL maintained this strategy, causing IC/VC to rise further; whereas other subjects averted EFL by subsequently raising end-expiratory lung volume back to near resting levels, such that IC/VC remained relatively stable despite rising exercise intensity. Indeed, ERV/VC was consistently higher (all *p*-values < 0.002) and IC/VC consistently lower in (all *p*-values < 0.003) non-EFL subjects once exercise began (Figure 1). The addition of an interaction term for EFL^∗^Work did not add significantly to the model fit (*p* = 0.27).

### Relationship to Symptoms

Prevalence of EFL among the three groups is shown in Table 3. EFL was less common in subjects with DoE compared with the asthma or “other” groups (*p* = 0.038). Dyspnea, alone or with leg fatigue, was cited by half the subjects as the reason for inability to continue at peak work, at similar rates in subjects with or without EFL (*p* = 0.80).

**Table 3:** Stated cause for exercise cessation among the three diagnostic groups.

EFL	No (*N* = 61)	Yes (*N* = 87)	Total (*N* = 148)	*p*-value
**Diagnostic group**				
DoE	43 (70%)	43 (49%)	86 (58%)	0.038^∗^
Asthma	11 (18%)	27 (31%)	38 (26%)	
Other	7 (12%)	17 (20%)	24 (16%)	
**Reason to stop**				
Leg fatigue	17 (28%)	20 (23%)	37 (25%)	0.6311
Dyspnea	24 (39%)	30 (35%)	54 (36%)	
Fatigue	11 (18%)	23 (26%)	34 (23%)	
Leg Fatigue +Dyspnea	9 (15%)	14 (16%)	23 (16%)	


*^∗^Pearson’s χ^*2*^ test.*

### MVV vs. FVL

**FIGURE 2:**
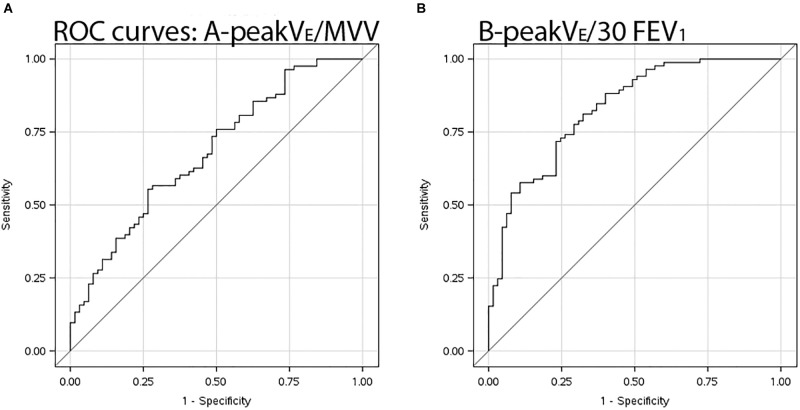
ROC curves. Receiver operating characteristic curves illustrating sensitivity and specificity of **(A)** 12-s sprint MVV in denominator for peak ${\dot{V}}_{E}$/MVV, and **(B)** proxy measure for MVV *viz.* peak ${\dot{V}}_{E}$/30⋅FEV_1_.

Maximum voluntary ventilation was similar in EFL vs. non-EFL subjects, but only slightly better than chance at identifying subjects who developed EFL during exercise with an AUC of only 0.69 (95% CI 0.61–0.78). AUC for multiples of FEV_1_ as surrogate measures for MVV are shown in Table 4. Having shown no difference in AUC among FEV_1_ multiples, Table 5 shows combinations of sensitivity and specificity for peak ${\dot{V}}_{E}$/30⋅FEV_1._ The optimal cut-point from the AUC (Figure 2) was 0.853 (95% CI 0.764–0.894), significantly better (*p* < 0.001) than MVV.

**Table 4:** Area under curve (AUC) results using MVV and different multiples of FEV_1_ in denominator.

Peak ${\dot{V}}_{E}$/*x*⋅FEV_1_ *x* =	AUC (95% CI)	Best cutoff to maximize sensitivity and specificity	Sensitivity	Specificity
0.30	0.840^∗^ (0.776, 0.905)	0.819	0.816	0.705
0.35	0.840	0.702	0.816	0.705
0.40	0.840	0.614	0.816	0.705
MVV	0.695 (0.614, 0.785)	0.850	0.623	0.617


*^∗^95% CI identical for different multiples of FEV_*1*_.*

**Table 5:** Exploration of cut-off values for peak ${\dot{V}}_{E}$/30 FEV_1_ compared to current interpretation standard.

Cut-point peak${\dot{V}}_{E}$/30⋅FEV_1_	Sensitivity (%)	Specificity (%)	PPV (%)	NPV (%)
>0.80	86	61	72	79
>0.85	76	78	80	74
>0.90	63	86	84	67
MVV^∗^	62	62	71	54


*PPV and NPV, positive and negative predictive values, respectively.*

*^∗^Using value > 0.850 for ratio of peak ${\dot{V}}_{E}$/MVV.*

## Discussion

Measured MVV was only marginally better than chance at identifying subjects with ventilatory limitation whereas FEV_1_ was superior to 12-s sprint MVV for estimation of maximum breathing capacity on exercise compared to EFL demonstrated by tidal FVL analysis. Use of MVV as the benchmark for maximal exercise is problematic in children. Healthy pre-pubertal children often achieve levels of peak ${\dot{V}}_{E}$ at or very close to MVV. Godfrey (Figure 4.2 of his book) speculated this observation was due to a mix of relatively high ventilatory requirements of younger children combined with their inability to properly perform an MVV maneuver (Godfrey, 1974). A recent study indeed found that 26% of children were unable to properly perform MVV maneuver (MacLean et al., 2016). ROC curve analysis confirmed that measured MVV is not particularly useful for concluding ventilatory limitation to exercise compared to FVL analysis. Ventilatory reserve is defined by peak exercise ${\dot{V}}_{E}$ as a fraction of MVV with the lower limit set at 15% (American Thoracic Society and American College of Chest Physicians, 2003). This cut-off is reasonable based on 95% confidence limits of adult norms but is not independent of fitness or aging (Ross, 2003). Newer textbooks on pediatric exercise medicine state “one can infer that ventilatory reserve increases with age, at least for males” (Bar-Or and Rowland, 2004); and children achieve “near to or slightly less than 70% (of MVV) at maximal ventilation” (Fawkner, 2007). We submit that tidal FVL demonstrating EFL is the preferred method for this designation, but one can be confident without said analysis if the ratio of peak ${\dot{V}}_{E}$/30⋅FEV_1_ exceeds 0.85. Our data adds wanting scientific rigor and validity to current interpretation standards. This index provides a target peak ${\dot{V}}_{E}$ from which one can judge maximal effort; is similar to ${\dot{V}}_{E}$ sustainable by adolescents during eucapnic voluntary hyperpnea (Van der Eycken et al., 2016); and to a surrogate measure for MVV in exercising CF patients (Stein et al., 2003).

### EFL and Ventilatory Limitation vs. Exercise Limitation

Numerous studies have been published using FVL analysis as a means of demonstrating ventilatory constraint to exercise despite limitations of the technique (Calverley and Koulouris, 2005). Exercise FVL have been used more often in adult studies but there is growing acceptance if not tacit recognition of its merits and limitations to detect EFL during exercise. EFL is common in pre-pubertal children (Nourry et al., 2006; Swain et al., 2010), obese adolescents (Gibson et al., 2014), and more common in trained vs. untrained pediatric subjects (Nourry et al., 2005); but its prevalence falls after puberty from 90 to 45% in boys and from 90 to 20% in girls (Emerson et al., 2015; Smith et al., 2015) whose mean ages ranged from 14 to 15 years. Dysanaptic lung growth may contribute to this changing prevalence of EFL (Smith et al., 2014). Nearly 60% of our subjects aged 14.3 ± 2.6 years had EFL during exercise indicating an enriched group with EFL among subjects evaluated for dyspnea. The incidence of EFL in males and females was ∼69 and 52%, respectively, which was higher than previously reported in the smaller cohort of post-pubescent adolescents (Emerson et al., 2015). This discrepancy is likely due to the inclusion of both pre, peri, and post- pubescent pediatric subjects in the present study; a caveat being gas compression artifact that may overestimate EFL (see below). In addition, we found that females that exhibited EFL had greater peak $\dot{V}O_{2}$, ventilation, and respiratory rate compared to females that did not exhibit EFL. These data suggest that girls who achieve higher workloads and metabolic rates are more likely to exhibit EFL during exercise similar to women (McClaran et al., 1998).

Mechanical constraints to ${\dot{V}}_{E}$ affect operating lung volume and breathing pattern during heavy exercise in subjects who experience EFL (McClaran et al., 1999). Babb postulated that onset of dynamic airway compression may be just as critical as EFL in evoking adjustments to minimize degree of EFL during exercise (Babb, 2013). Such strategic changes may demand more perfusion of the respiratory muscles potentially depriving working leg muscles (Harms et al., 1997), and leg muscle fatigue resulting from high-intensity exercise is at least partly due to increased inspiratory muscle work (Dempsey et al., 2006). Unloading the respiratory muscles by Heliox breathing resulted in small but statistically significant boost in performance at heavy exercise (Wilkie et al., 2015); whereas mechanical unloading with proportional-assist ventilation resulted in clinically significant improvement in performance at 90% peak $\dot{V}O_{2}$ (Harms et al., 2000). Hyperinflation attenuates stroke volume at rest and response to exercise in healthy controls (Stark-Leyva et al., 2004; Cheyne et al., 2018). There is likely a hierarchy of blood flow distribution during exercise between respiratory vs. locomotor musculatures and muscle afferent feedback influence fatigue and dyspnea (Sheel et al., 2018).

### Exertional Dyspnea

Half the subjects cited breathing as the reason for exercise test cessation though no particular limiting symptom was cited more often among those with vs. without EFL. Some subjects continued to exercise despite developing EFL before finally stopping but one should not be surprised by an uncoupling ventilatory constraint and exercise limited *by* dyspnea. The most likely explanation is that some subjects raised end-expiratory lung volume during heavy exercise to counter impending or evolving EFL, which would have generated greater elastic work of breathing as lung compliance falls at higher lung volumes. One might then cease exercise due to dyspnea *without* EFL manifest. Subjects in this report chose their breathing strategy early during exercise, in virtually all subjects *before* EFL developed. Examination of Figure 2 suggests that subsequent behavior may perhaps have mitigated EFL but those with EFL clearly had lower end-expiratory lung volume than did those without EFL by heavy exercise. Results of studies in children offer disparate findings with respect to dyspnea in presence vs. absence of EFL (cf. Nourry et al., 2006; Swain et al., 2010) and studies in adults show similar discordance (Lovering et al., 2014; Wilkie et al., 2015). The reality is that our understanding of mechanisms of dyspnea in pediatrics is rudimentary (Pianosi, 2018a).

### Clinical Significance

Triage of exertional dyspnea in pediatric populations is hindered by lack of data, forcing clinicians to rely on empiricism derived from adult subjects. Abu-Hasan and Weinberger concluded respiratory limitation from “restrictive” physiology as responsible if a subject’s exercise ventilation fell within specified ranges of tidal volume, breath rate, and breathing reserve, based on extrapolation from adult studies (Abu-Hasan et al., 2005). Mahut et al. (2014) similarly concluded that the most frequent cause of exertional dyspnea in adolescents was “physiologic,” defined as normal aerobic power and ventilatory response. The present study creates a novel paradigm for attribution of exertional dyspnea in pediatric subjects using data obtained *from* pediatric subjects and suggests a possible explanation. Just as Dominelli et al. (2015) stated some healthy individuals are more likely to exhibit mechanical constraints during exercise, one may postulate the respiratory system is culpable in pediatric subjects evaluated for exertional dyspnea, particularly females who push themselves (Table 2). We believe it is no coincidence that exercise enhances every component of the O_2_ transport system except the lungs. Ergo, the lungs *may* contribute to the limitation of peak $\dot{V}O_{2}$ (Wagner, 2005).

### Limitations

There are caveats when using exercise tidal FVL to determine EFL (Guenette et al., 2010, 2013; Dominelli and Sheel, 2012). First, among alternative methods, only negative expiratory pressure (NEP) technique has been employed in pediatrics – and only in infants (Jones et al., 2000). We did not measure IC at peak exercise, meaning placement of tidal breaths at peak exercise was based on IC maneuvers done at work up to 90% peak work. However, Nourry et al. (2006) showed minimal change in end-expiratory lung volume from this range to peak work. Second, the best acceptable maximum flow-volume curve at rest (pre-exercise) was used to place the exercise FVL within its maximum envelope. Swain et al. (2010) noted only ∼3% difference between pre- and post-test maximum expiratory flow-volume curves. We essentially doubled this inherent variability when choosing our EFL threshold. Moreover, said factors could affect determination of EFL in *either* direction, and any bias should be nullified given the sample size, as these pitfalls could positively *or* negatively influence EFL adjudication. The effect of thoracic gas compression on FVLs when volume is measured at the mouth may affect identification of EFL during exercise, but there are no data concerning this in children and adolescents; and any presumption that adult studies apply to children or adolescents should be viewed with skepticism in view of the high prevalence of EFL in children. Though certainly present, we truly do not know the volume gas compression in pediatric subjects during exercise. During routine spirometry, its magnitude is unpredictable (Coates et al., 1988) but, with few exceptions, one would expect it to be small in the population reported herein, most of whom had normal spirometry. Our subject sample did not include patients with cystic fibrosis. Indeed, Stein et al. (2003) proposed an equation for calculating MVV based on FEV_1_ in CF patients which performed nearly as well as an FEV_1_-based proxy measure, and which is very similar to our selection of 30⋅FEV_1_. Finally, dyspnea ratings were not recorded because existing dyspnea scales had dubious validity in children when testing began in 2007. We recently published our validation studies in pediatric subjects (Pianosi et al., 2014, 2015), and found remarkable similarity in dyspnea ratings during exercise among healthy controls, subjects with asthma, or cystic fibrosis.

## Conclusion

30⋅FEV_1_ is superior to MVV for assessing potential ventilatory limitation to exercise defined as >5% overlap of exercise (tidal FVL with maximal expiratory FVL) in pediatric subjects if tidal FVL analysis is unavailable and could be used to decide whether a child or adolescent experiences exercise limitation due to the respiratory pump. ROC analysis for breathing reserve < 0.15 yields positive predictive value of nearly 80% and negative predictive value of 75% for detecting EFL.

### Future Directions

Defining EFL is still a moving target which will be subject to change as our understanding of, and limitations to, using tidal FVL analysis to determine ventilatory limitation. There are caveats when using exercise tidal FVL to determine EFL, chief among which are understanding how thoracic gas compression during a forced expiratory maneuver or during active exhalation such as may occur during heavy exercise may affect flow rate at any given lung volume, so more research is necessary in this domain. Ideally, this should be explored by plotting iso-volume pressure-flow diagrams, or employing NEP technique (Eltayara et al., 1996), to quantify how thoracic gas compression affects FVL during exercise in pediatric subjects.

## Ethics Statement

The study meets the guidelines for ethical conduct and report of research, and was approved the Mayo Clinic IRB study ID# 14-009335.

## Author Contributions

PP supervised exercise testing, collated and data, and wrote initial draft. JS revised the manuscript.

## Conflict of Interest Statement

The authors declare that the research was conducted in the absence of any commercial or financial relationships that could be construed as a potential conflict of interest.
